# Case of Nigeria-Acquired Human African Trypanosomiasis in United Kingdom, 2016

**DOI:** 10.3201/eid2307.170695

**Published:** 2017-07

**Authors:** Akish Luintel, Patricia Lowe, Anneli Cooper, Annette MacLeod, Philippe Büscher, Tim Brooks, Mike Brown

**Affiliations:** University College London Hospital, London, UK (A. Luintel, P. Lowe, M. Brown);; University of Glasgow, Glasgow, Scotland, UK (A. Cooper, A. MacLeod);; Institute of Tropical Medicine, Antwerp, Belgium (P. Büscher);; Public Health England, London (T. Brooks);; London School of Hygiene and Tropical Medicine, London (M. Brown)

**Keywords:** African trypanosomiasis, Nigeria, Africa, United Kingdom, sleeping sickness, parasites, protozoa, zoonoses, human, Trypanosoma brucei gambiense, surveillance, vector-borne infections, tsetse fly

## Abstract

Human African trypanosomiasis has not been reported in Nigeria since 2012. Nevertheless, limitations of current surveillance programs mean that undetected infections may persist. We report a recent case of stage 2 trypanosomiasis caused by *Trypanosoma brucei gambiense* acquired in Nigeria and imported into the United Kingdom.

Human African trypanosomiasis (HAT), known as African sleeping sickness, is a protozoal infection, the West African form of which is caused by *Trypanosoma brucei gambiense*. We report a case of imported *T. brucei gambiense* HAT, acquired in Nigeria, where no cases have been reported since 2012 (*1*).

The case-patient, a 58-year-old Nigerian woman, lived near Warri, in Delta State, Nigeria. She traveled infrequently to towns within Delta State, across the Niger River into Bayelsa State, and to larger cities in Nigeria, but never outside Nigeria. She reported no history of tsetse fly bites.

In January 2016, the patient experienced leg tremors and lethargy. These symptoms persisted until arrival in the United Kingdom in May 2016. Over the next 2 months, increasing malaise and unsteadiness in walking developed. In August 2016, the patient was admitted to a regional hospital with confusion and drowsiness. She was febrile at admission but had no lymphadenopathy; neurologic examination revealed no neck stiffness or photophobia, but did show poor coordination with slow cognitive processes.

Laboratory investigations revealed microcytic anemia with a C-reactive protein level of 13 mg/L (reference value <5 mg/L) and a total serum IgM of 13.7 g/L (reference range 0.5–2.0 g/L). A blood film was negative for malaria. Confirmatory assays after positive screening assay results for HIV and syphilis antibodies showed the original results to be false positive.

Cerebrospinal fluid (CSF) examination revealed 331 leukocytes/mm^3^, 99% lymphocytes; CSF protein level 0.82 g/L (reference range 0.23–0.38 g/L); and glucose level was >50% plasma glucose. Results of CSF PCR for herpesviruses, enterovirus, and JC virus were negative. Results of GeneXpert (Cepheid, Buckinghamshire, UK) tests of CSF and mycobacterial culture were negative. Magnetic resonance imaging of the brain showed, on T2 weighted and flair images, bilateral diffuse hyperintensities within white matter located in the periventricular regions, basal ganglia, cerebellum, and brainstem.

Treatment with ceftriaxone, acyclovir, antituberculous treatment and prednisolone was stopped at 14 days because of a lack of clinical improvement and drug-induced transaminitis.

The patient’s lethargy, intermittent confusion, and periods of somnolence became more severe. Examination in August 2016 revealed intention tremor in all limbs and myoclonic jerks. Her case was discussed with the Imported Fever Service at Public Health England. A serum sample was sent to the Hospital of Tropical Diseases in London, UK for *T. brucei gambiense* indirect fluorescent antibody testing (IFAT), which showed a positive result (titer 1:400). The patient was transferred to this hospital.

Repeat CSF examination revealed a protein level of 1.14 g/L, CSF glucose level of 2.3 mmol/L (serum 5.5 mmol/L), and 1,140 leukocytes/mm^3^ (90% mononuclear). No trypanosomes were seen in the buffy coat of peripheral blood or CSF. CSF total IgM of 1.98 mg/L (reference range 0–0.9 mg/L) and IgG of 306 mg/L (reference range 10–40 mg/L) were markedly raised (*2*).

*T. brucei gambiense* IFAT results for CSF (titer 1:4) and blood (titer 1:400) were positive. DNA extracted from CSF was positive for trypanosomes of the subgenus *Trypanozoon* by PCR (*3**)* and confirmed as *T. brucei gambiense* group 1 by diagnostic PCR with *TgsGP* primers (*4*)*.* The result of immune trypanolysis was negative when *T. brucei gambiense* variable antigen type LiTat 1.3 was used but positive when using LiTat 1.5 (*4*). The patient had a titer of 1:32 in the CATT/*T. brucei gambiense* test (*5**,**6*).

We administered nifurtimox/eflornithine combination therapy, according to World Health Organization guidelines, for treatment of stage 2 *T. brucei gambiense*. By the end of treatment, the patient’s cognitive function, myoclonus, tremor, and ataxia had all improved, and she was discharged.

A posttreatment CSF examination revealed 4 leukocytes/mm^3^ and a protein level of 1.17 g/L. Six months after discharge, the patient had made a full functional recovery. Repeat CSF examination revealed 4 leukocytes/mm^3^ and a protein level of 0.63 g/L, and MRI of the brain showed improvement. The CSF *T. brucei gambiense* IFAT titer declined to 1:2.

No cases of HAT in Nigeria have been reported to the World Health Organization since 2012 (*1*). The last reported patients were from the Abraka region, in Delta State, ≈50 km from this case-patient’s residence (*7*)*.* We do not have a clear exposure time for this patient, which is likely to have been within 2–3 years before her hospitalization, although we have reported a case-patient with an exposure >29 years prior to illness onset; the emerging scientific consensus is that trypanotolerance may exist among some infected patients, leading to delay in diagnosis and premature confidence of disease elimination (*8**,**9*).

A clue to diagnosis, alongside clinical and travel history, was a high serum IgM level, a prominent feature in *T. brucei gambiense*–infected patients, as a result of polyclonal B-cell activation (*10*). False-positive antibody test results specific for pathogens have been reported; multiple positive antibody tests may aid the clinician in considering HAT as a differential diagnosis (*10*).

This case shows that HAT transmission continues in Nigeria and surveillance in Delta state is warranted. There may be a role for additional diagnostic tools if we are to achieve the goal of eliminating HAT by 2030.
